# Application of Bacteriophages in Nanotechnology

**DOI:** 10.3390/nano10101944

**Published:** 2020-09-29

**Authors:** Jan Paczesny, Krzysztof Bielec

**Affiliations:** Institute of Physical Chemistry of the Polish Academy of Sciences, Kasprzaka 44/52, 01-224 Warsaw, Poland; kbielec@ichf.edu.pl

**Keywords:** bacteriophages, phage display, phage therapies, phage-based sensors, soft matter, materials, scaffolds, viruses

## Abstract

Bacteriophages (phages for short) are viruses, which have bacteria as hosts. The single phage body virion, is a colloidal particle, often possessing a dipole moment. As such, phages were used as perfectly monodisperse systems to study various physicochemical phenomena (e.g., transport or sedimentation in complex fluids), or in the material science (e.g., as scaffolds). Nevertheless, phages also execute the life cycle to multiply and produce progeny virions. Upon completion of the life cycle of phages, the host cells are usually destroyed. Natural abilities to bind to and kill bacteria were a starting point for utilizing phages in phage therapies (i.e., medical treatments that use phages to fight bacterial infections) and for bacteria detection. Numerous applications of phages became possible thanks to phage display—a method connecting the phenotype and genotype, which allows for selecting specific peptides or proteins with affinity to a given target. Here, we review the application of bacteriophages in nanoscience, emphasizing bio-related applications, material science, soft matter research, and physical chemistry.

## 1. Introduction

Nanoscience is “enabling technology”, which impacts numerous fields of research and everyday life. The socio-economic implications of introducing new nanotechnology or a class of nanomaterials can be, therefore, far-reaching (across many economic sectors) and very deep (starting at the beginning of the value/supply chain) [1]. The most widely used in the industry is the “top-down” approach for the preparation of nano-objects. “Top-down” utilizes external tools and forces the creation of smaller objects from larger entities (via e.g., milling, lithography, printing). It seems that the developments in the field are reaching their limit, with Intel not being able to master a 7 nm process for many years now [2]. Alternatively, in the “bottom-up” approach, individual parts self-assemble spontaneously to form ordered and/or functional structures due to specific interactions, often imposed on these building blocks. Despite all the advances that nanotechnology has brought to us, the abiotic self-assembling systems are relatively simple, and their functionalities are restricted. Even with the introduction of dynamic self-assembly, which allowed to design systems consuming energy to maintain their structure and functions [3], and some advantages in creating chemical networks [4], we are still far behind the level of complexity present in nature. Nevertheless, we have learned to take advantage of biotic functional systems and incorporate some “natural” building blocks into human-made nanodesigns. Here, we review the specific manifestation of such an approach, namely the utilization of bacteriophages (phages for short), i.e., viruses, which have bacteria as hosts.

The average size of the virion, i.e., a single phage body, is around 30–200 nm, but the largest might be more than 800 nm in length [5]. The most popular phages fit perfectly into the category of nano-objects, i.e., having at least one geometrical dimension smaller than 100 nm. Nature offers a great variety of the possible structural morphologies of phages. The majority belong to the order of *Caudovirales* and share a typical structure design, i.e., the genetic information is stored in a capsid, to which a spike-tail with fibers is attached [6,7]. Less common are filamentous (e.g., M13) or nearly spherical (isometric) phages (e.g., MS2). Filamentous phages are broadly used, as they are most robust in phage display (cf. Section 2.1), and they form liquid crystalline phases (cf. Section 4). MS2 is used as a model of eukaryotic viruses in a number of bio-related studies [8]. T1, T4, T7, or Lambda phages are the most well known examples of tailed phages and are often chosen as model systems representing the most abundant *Caudovirales* order. It is estimated that there are around 10^31^ phages on the planet, making them the most abundant and diverse biological entities. They have a profound role as modulators in numerous biomes, ranging from the human gut to biogeochemical cycling in aquatic environments [7].

Phages can be produced easily and cheaply in large quantities and easily purified. By only infecting a bacteria solution, one can obtain a large number of progeny phages. Phages undergo evolution, and thus they remain effective against bacteria [9]. For example, anti-CRISPR (Anti-Clustered Regularly Interspaced Short Palindromic Repeats) [10] mechanism was discovered shortly after CRISPR [11]—a molecular mechanism that allows bacteria to recognize and degrade alien genetic material. Some phages are robust and retain their activity even after exposure to high temperatures [12], pH [13], and organic solvents [14,15]. Each virion of a given phage is identical (no polydispersity of sizes), whereas chemical and genetic modifications allow for the preparation of the virions of desired properties. All these traits make phages exciting building blocks for utilization in nanotechnology.

## 2. Bacteriophages in Bio-Related Applications

**Figure 1:**
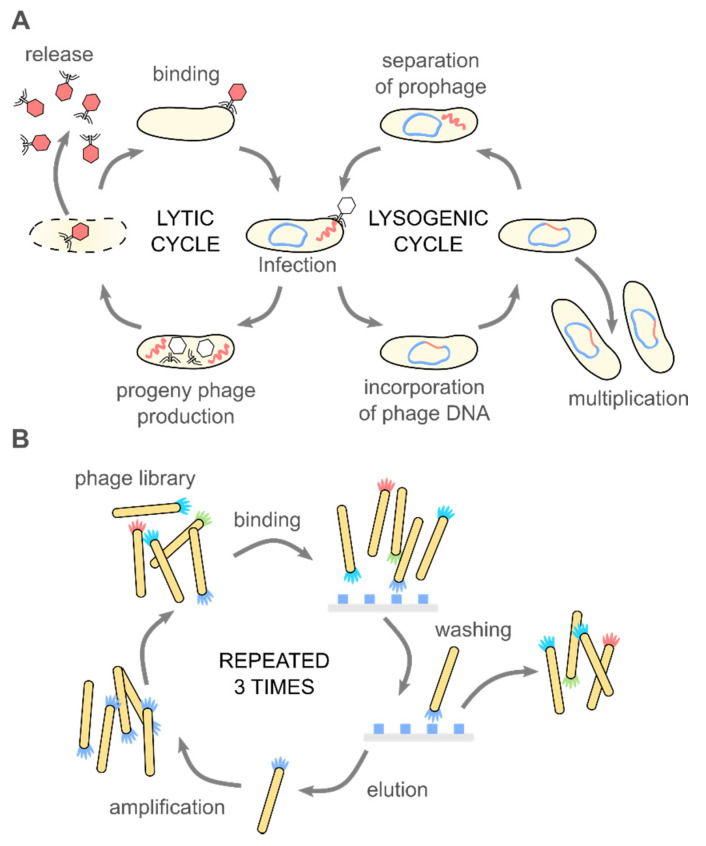
(**A**) Bacteriophages are obligate parasites, which usurp the molecular machinery of the host to complete their life cycle. First, phage virions bind to target bacteria and inject the genetic information into the host cell. Only the recognition of a proper host assures the completion of the cycle. In the case of lytic phages, progeny virions are produced inside the host cell. The release of the progeny virions usually results in the disruption and death of the bacterium. Temperate phages integrate their genetic information with the genome of the host, forming a prophage. A prophage replicates upon divisions of the bacteria. External stimuli (e.g., food shortage, UV light, temperature) might activate the prophage, and thus start the lytic cycle. These natural mechanisms (i.e., binding to specific target bacteria and amplification), along with the monodispersity of virions and a variety of morphologies, are fundamental for the application of phages in phage therapies, biocontrol, sensing, material science, and soft matter research. (**B**) The discovery of the phage display method significantly broadened the possible usage of bacteriophage. In the phage display, a fusion of large libraries with genes coding coat proteins allows for a direct correlation between the genome and phenotype. Bio-panning allows for selecting specific peptides or proteins with affinity to a given target. These selected peptides might be used separately or provide phage virions with additional functionalities. Phage display was crucial in the development of new drugs, vaccines, but also material science and physical chemistry.

Phages attach to the host cell and introduce genetic materials inside bacteria. What happens next determines the qualification of bacteriophages as a lysogenic and lytic one (see Figure 1A). In the lytic cycle, a host’s cell is disrupted to free progeny phages from the infected bacteria. Lysis is possible due to the amurins, which are proteins that inhibit peptidoglycan synthesis [16]. In the lysogenic cycle, the viral genome integrates into the chromosome of bacteria and remains latent, replicating for generations [17]. When viral genetic material is incorporated into the chromosomal DNA of bacteria, it is known as a prophage [18]. The appearance of stressors, e.g., chemicals, UV radiation, or damage of the host DNA, can cause the conversion of the cycle and change from lysogenic into the lytic [19]. Only some filamentous phages might cause the continuous generation of progeny virions without causing the death of the host [20]. Bacteriophages are considered non-toxic to eukaryotes because structural elements of the virion cannot bind to eukaryotic cells [21].

### 2.1. Phage Display and Phage-Based Delivery Systems

One half of the 2018 Nobel Prize in Chemistry was awarded to George P. Smith and Sir Gregory P. Winter “for the phage display of peptides and antibodies” [22]. The method utilizes the possibility to genetically modify phages to display peptides, proteins, or antibodies at the surface via fusion with appropriate gene product. This results in a connection between genotype and phenotype. The exposition of phages possessing various inserts to a target material allows for the screening of large libraries in search of peptides or even proteins that interact with the target. Bio-panning allows for selecting specific peptides or proteins with affinity to a given target (see Figure 1B). Biopanning involves four major steps: (1) the preparation of phage display libraries, (2) capturing steps, where virions displaying sequence having affinity to the target bind to it, (3) washing step, which removes unbound virions, and (4) the elution step, which allows collecting phages with specific affinity. In the earliest examples, genes were cloned directly into the phage genome. Alternatively, phagemid vectors are used; in such a case, “helper phage” is needed to produce functional virions. The M13 phage, and the closely related fd and f1, are the most extensively used in phage display [23].

The possibility to probe and identify ligand–receptor interactions allowed for advancement in studies of infectious diseases [24] and cancer [25,26]. This resulted in improvements in drug discovery and vaccine design [27]. The overview of the phage-display method and its utilization in medicine can be found in recent reviews by Mimmi et al. [28], Sokullu et al. [29], Petrenko [30], Garg [31], Sunderland et al. [32], and Newman and Benoit [33].

In a new example by Lauster and coworkers [34], the modification of a phage capsid with non-natural amino acid (L-homopropargylglycine) was introduced to the coat protein for spatial control over the distribution of ligands binding to the spike protein of the influenza A virus. The appropriate geometrical distribution of ligands was possible due to the symmetric, icosahedral structure of bacteriophages Qβ. These capsids bind to the influenza A virus envelope in the multivalent mode, thus inhibiting the infection of eukaryotic cells.

Not only medicine but also nanotechnology are taking advantage of the phage-display technique. There are examples of phage display-selected peptides for the binding of nanoparticles (e.g., noble metals [35], ZnO [36], Fe_3_O_4_ [37,38], semiconductors TiO_2_, CdS, ZnS, (SiO_2_) [39,40,41,42,43]), minerals [44] or ions (e.g., arsenic (III) [45]). The potential applications vary from sensing [45,46,47], separation, and processing [44] to templated nanoparticle synthesis, where phage display-selected peptides control the nucleation and growth of inorganic nanoparticles [48,49].

Bacteriophages are also utilized as delivery carriers [50]. Displaying specific peptides targeting eukaryotic cells or tissues compensates for the lack of natural mechanisms to enter mammalian cells or reach appropriate intracellular compartments. Non-modified phages are sometimes used, but the efficiency of virions containing cell-specific targeting peptides developed by phage display [51] or even phage-inspired nano-carriers [52] is superior. There is a possibility of coupling a variety of loads (e.g., drugs) by genetic manipulation [53,54] or chemical conjugation [55,56]. Upon the recognition of the target, payloads might be released in a controlled manner. Hyman said that even despite the size of phages, they “only very loosely fit into the nanotechnology category” [57]. Therefore, here we focused on chemically modified systems and artificial designs.

Lambda phage was used as early as 1971 to deliver the gene for galactose transferase into human fibroblast cells isolated from a patient with a deficiency in this enzyme [58]. M13 was used for the targeted delivery of the GFP gene-expression cassette into breast cancer cells [59]. Filamentous phage f1 with targeting antibodies displayed on the tip of the virion was used as a carrier of chemically conjugated antibiotics [60]. Authors showed that such nanomedicine is non-toxic to mice, its immunogenicity is reduced in comparison to native phages, whereas the half-life of the conjugated phage increased in the bloodstream. Icosahedral MS2 phage might undergo genetic manipulation, chemical conjugation, and the removal of genetic material resulting in an empty nano-carrier, which might be loaded with the cargo of choice. For instance, Stephanopoulos et al. [61] showed the dual modified virus capsids: the cell-specific aptamer was conjugated to the unnatural amino acids (p-aminophenylalanine) displayed at the outer surface, whereas porphyrins were attached to the specially introduced cysteine residues at the inner surface. Upon the illumination of porphyrins, singlet oxygen was generated, which led to the selective destruction of target cells.

Recently, Zhu et al. [62] showed a prokaryotic–eukaryotic hybrid, composed of T4 phage and adeno-associated virus (AAV). The bacteriophage acted as the cargo, which might be reasonably easily modified. Th eukaryotic virus acted as a “driver” that allowed for cargo delivery into mammalian cells. The authors showed the example of the hybrid vector, where double stranded luciferase plasmid was packed into a T4 capsid, β-galactosidase was displayed at the phage capsid, and additionally, single stranded GFP DNA was packed in AAV capsid. This load was delivered simultaneously into the cell. The luciferase activity was four orders of magnitude larger compared to only T4 virions.

Phage proteins themselves can also assemble into nano-carriers for gene delivery [52]. For instance, the Petrenko group showed the encapsulation of siRNA by fused protein composed of phage capsid protein and targeting moiety [63]. The phage-mimetic nanoparticle was named “nanophage” as it comprises both protein shell and genetic material stored inside. “Nanophages” were used to deliver siRNA to the target region and cause gene silencing.

Phage-displayed peptides were also used to create artificial, nanoparticulate delivery systems. For instance, chitosan nanoparticles were conjugates with a phage-display selected sequence targeting the follicle-associated epithelium region of Peyer’s patch [64]. This work is an example of a potential carrier for vaccine delivery. Other polymer-based nanoparticles, namely polyethylene glycol–poly(lactic-co-glycolic acid) (PEG–PLGA), modified with phage-displayed peptides, were used to target the brain [65]. The possibility to deliver drugs through the blood–brain barrier might impact the therapy of many central nervous system diseases. Interestingly, Ma et al. [66] showed the utilization of liposome modified with a specific phage-displayed peptide and loaded with DNA to target cell nuclei. The authors delivered the transposon system, which allowed the efficient insertion of transgenes into the host genome, into the nuclei of rat mesenchymal stem cells. Such a non-viral gene delivery vector is promising for stem cell therapy. Jin et al. [67] found a series of peptides prolonging the blood residence time for M13 bacteriophage. Later, they transferred this property to self-assembled heavy-chain ferritin nanocages. In addition, metal nanoparticles were decorated with specific, phage-displayed peptides. For instance, Wang et al. [68] developed Au@Ag heterogenous nanorods, which were directed by fusion proteins into cancer cells, where metallic nanostructures were used for photothermal ablation. In a similar example, Peng et al. [69] conjugated gold nanorods with chimeric phages that were engineered to specifically attach to several Gram-negative bacteria. Upon excitation by near-infrared light, gold nanorods release energy locally, generating heat that efficiently kills targeted cells.

In a different approach, bacteriophages were used as a molecular label [70]. The information was included in the DNA, which was later read using PCR-driven amplification. Bacteriophages were applied to label a nanoscaled bulk material, e.g., multi-walled carbon nanotubes.

### 2.2. Phages as Antibacterial Agents: Phage Therapy, Biocontrol Applications

The intrinsic efficiency of phages against bacteria resulted in the development of phage-derived antibacterials [71]. Bacteriophages have been used for medical purposes since the early 1900s [17,72]. Phages can support the development of inflammatory response against bacteria via the lysis of bacterial cell wall, which activates the immune system [73]. Therefore, phage therapies, i.e., the administration of phage cocktails to patients infected with bacteria, besides the direct elimination of bacteria cells, additionally activate the human immune system to fight against infection. Phage therapies were largely abandoned when antibiotics were developed [74]. However, the spread of drug-resistant superbugs and the lack of new medicines [75,76] has caused a renaissance of bacteriophage-based antimicrobials [77]. Phages are therapeutically used to treat bacterial infections that do not respond to conventional antibiotics, particularly in Russia and Georgia [78]. In these countries, phage products are directly available, even without a prescription (e.g., “*Intestiphage*”) [79]. The Russian company Microgen sells phages as liquid preparations or as pills [80]. In recent years, phage-based treatments reached clinical trials, e.g., curing inner ear infections [81], typhoid [82], or infected burn wounds (“*Phagoburn”* project) [83]. The “*Phagoburn”* project was terminated early because the utilized phage cocktails were proved to not be effective [83]. In 2019, the Food and Drug Administration approved the first clinical trial in the USA for intravenous phage therapy [84]. The very compelling reviews on the history of phage therapies are given by Abedon et al. [79] and Cisek et al. [16]. The summary of the current situation is given by Altamirano and Barr [85].

The ability of phages to kill target bacteria is also used in biocontrol applications, e.g., in the food industry and agriculture. The application of phages is safer compared to the utilization of antibiotics [86]. In 2017, the European Commission adopted the “EU One Health action plan against antimicrobial resistance”. It was the first step toward the control of the use of antibiotics in agro-food production, as it is a major source of bacterial resistance acquisition [87]. Phages were used to protect dairy products [88], fruits [89], vegetables [90], meat [91], and fish [92]. Developments in the utilization of phages as antimicrobial agents in plant and animal agriculture at the farm level are summarized in a recent review by Svircev et al. [93].

## 3. Bacteriophages in Materials Science

**Figure 2:**
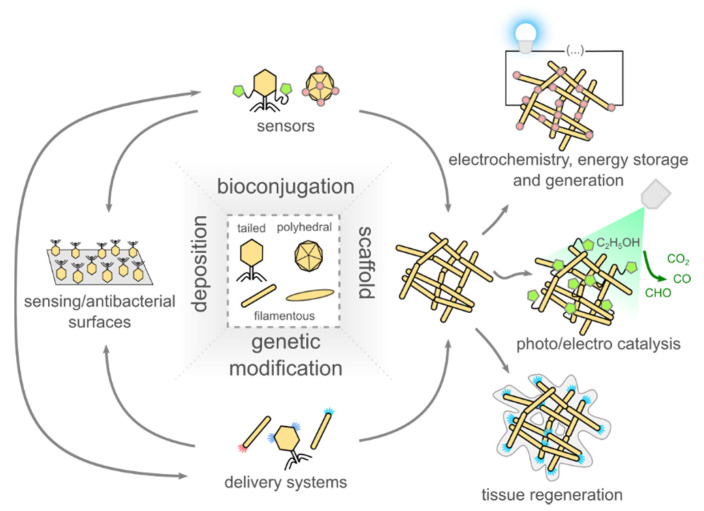
Scheme showing the main fields of application of bacteriophages in materials science. The main approaches are: (1) the utilization of the ability of phages to capture and infect bacteria, (2) taking advantage of the ease of virions modification, both by chemical and genetic means, and (3) using virions to produce scaffolds, later used, for example, in electrochemistry, energy storage, and generation application, for the production of catalysts and for tissue regeneration.

The use of bacteriophages in sciences related to biology is an indigenous process. On the other hand, the application of phages in materials, composites, or even micro-robotics seems promising [57,94,95]. The nanometer virus structure is monodisperse and in the case of some phages, self-assembling [96,97]. Those features, followed by the rapid multiplication of uniform copies with a previously selected or even genetically engineered structure, are ideal for applying phages either as building components or templates/scaffolds for the production of novel bio/nanomaterials [98,99,100,101,102]. The physicochemical properties of individual phages determine the future use of phage-based nanomaterials. Phage resistance to temperature, solvent, or pH can considerably vary from one type to another [103,104]. In other examples, virions might be manipulated by the external stimuli, e.g., thanks to the permanent dipole moment [105,106]. Often, the usability of the given phage is directly related to the stability of the capsid, which may have been solvated or denatured under the operational conditions of the final material [107,108]. The overview of most prominent phage-related studies in the field of material science is shown in Figure 2.

**Figure 3:**
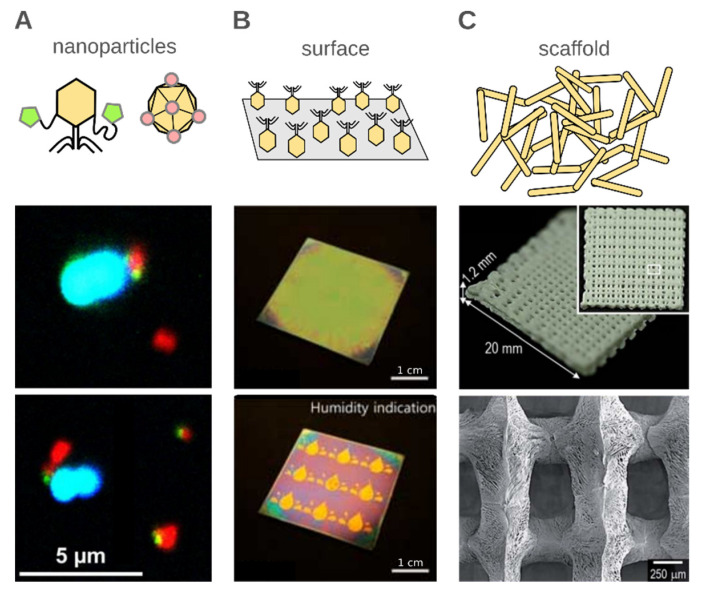
(**A**) Example of bioconjugates composed of multifunctional (magnetic and fluorescent) particles (red) and bacteriophages (stained green) used for the detection of bacteria (stained blue) using flow cytometry. Adapted with permission from reference [109]. Copyright 2017 American Chemical Society. (**B**) Phages deposited at the surface are often (but not exclusively) used for the preparation of antibacterial packaging or for sensing. Here, we demonstrate an example where the M13 phage layer rapidly responds to external factors (various volatile organic chemicals and endocrine-disrupting chemicals), displaying colorimetric behavior. Adapted from reference [112] based on the Creative Commons Attribution License (CC BY 4.0). (**C**) The monodispersity of virions and a variety of morphologies make phages great building blocks for scaffolds. Here, we show an example where a scaffold utilizing M13 phages and polymers (alginate and poly(ε-caprolactone)) was suggested for use in bone tissue regeneration. Adapted from reference [113] with permission from the Royal Society of Chemistry.

Bacteriophages are used in connection to 1D (nanoparticles), 2D (surface layers) and 3D (scaffolds) materials (Figure 3). In addition, three main approaches to synthesizing phage-based materials can be distinguished. First, the phage structure is unchanged, and usually, phages are still viable (e.g., the preparation of multifunctional conjugates composed of abiotic functional particles and phages [109], see Figure 3A). In such a case, the resultant materials often have applications in bio- or medicine-related applications [110]. The synthesis of such materials is usually done in water-based buffers and at a temperature close to room temperature. The second type of technological process exploits the nanometer dimensions of phages to create scaffolds or templates apart from their biological properties. For example, in an example of TiO_2_ porous material synthesis, phages are mixed with titanium isopropoxide/ethanol based solution (50% *v*/*v*) and calcinated at 500 °C [98]. The last type of phage-based materials uses selected phage building components as the empty capsid, structural proteins, receptors, or displayed peptides. Delivery systems or nanoparticles using specific, phage-displayed peptides were shown (cf. Section 2.1), but also, for example, Lee et al. [111] reviewed the possible applications of the bacteriophage Phi29 DNA packaging motor in nanotechnology and therapy.

### 3.1. Antibacterial Materials

Phages are used as antibacterial agents not only for phage therapies or phage control applications but also to prepare novel, nanostructured materials [114], i.e., wound dressings [115], packaging [116,117] or a membrane for water treatment [118], reducing the risk of bacterial infections. There are recent examples of combining phages with synthetic [119] and natural polymers (e.g., chitosan [120,121], alginates [91], collagen fibers [122]), even in the form of electrospun fibrous mats [123]. As phages are not harmful to humans, it is possible to create edible and antibacterial materials [92,124,125]. Leppänen and coworkers showed the immobilization of flavobacterium infecting bacteriophage at the surface of silicon and gold, both using bare surface and chemically modified surfaces [126]. Interestingly, the authors suggested how to measure the efficiency of such composites—they proposed to use “effective phage forming units (PFU)/surface area”, as a comparable standard between different studies. Effective PFU is assessed by comparing the reduction in bacterial growth produced by the phage-modified materials to the effect caused by a known number of free phages.

Phage virions were also used as stabilizing agents for the synthesis of gold nanoparticles, which have antibacterial and antibiofilm properties [127]. Hopf et al. [128] went further—they synthetized abiotic nanoparticles, with a structure was similar to viruses, and named them phage-mimicking antibacterial core-shell nanoparticles. However, in this case, the antibacterial properties were mainly derived from silver coating, which was deposited in an anisotropic fashion on the gold nanospheres, which earlier were conjugated to relatively large silica particles.

Tolba et al. [129] used recombinant bacteriophages (bearing biotin carboxyl carrier protein gene or the cellulose-binding module gene) for the oriented immobilization of bacteriophage T4 at streptavidin-coated magnetic beads and cellulose-based materials. Later, Richter and coworkers [105,106] showed the ordered deposition of a T4 phage onto a gold surface (and a chemically modified gold surface). The authors utilized a large dipole moment of virions to orient them in the external electric field. Such an approach allowed more receptor binding proteins to be exposed and available for interactions with the bacteria. In the case of random deposition, steric hindrances decreased the activity of phage layers.

There might be additional benefits derived from the utilization of phage-modified materials. Meyer et al. [130] showed the stabilization of T4 virions deposited onto papers in comparison to free phages against extreme pH. Leung et al. showed the preservation of the antimicrobial activity of P100, CG4, and AG10 bacteriophages in sugar (pullulan–trehalose mixture) as dried films and as coatings on food packaging [131,132]. In addition, Kimmelshue showed the prolonged storage time of CN8 phages when polyvinyl alcohol was used [119]. Fixed Phage Ltd. (Glasgow, UK) is developing both spray-based methods as well as films and biomaterials, having antibacterial properties for use in agriculture, food, veterinary, and personal health.

### 3.2. Biosensors

The natural ability of phages to capture host bacteria was used to develop phage-based methods for bacteria detection. Only the recognition of a proper and viable host assures the multiplication of virions and the completion of the life cycle of bacteriophages. This gives the advantage over other broadly used methods, e.g., PCR or mass spectroscopy-based, which are prone to false positives in case of the presence of dead bacteria. Compared to antibodies, phages are much cheaper and easier to prepare, whereas standard biochemical or culturing methods require a much longer time to generate an outcome [133,134].

There are two main designs of phage-based biosensors [133,134,135,136,137]. Methods, where the analytical signal is generated upon bacteria capturing (at the surface of the sensing elements or by phage-based probes), are fast, but a single event is difficult to detect, and thus the limits of detection (LOD) are relatively high. In contrast, infecting bacteria and utilizing their molecular machinery to amplify the signal (progeny virions, products of reporter genes, or metabolites) offers low detection limits, but the procedures are time consuming. There is still a need to develop methods allowing for the LOD in a range below 10 colony forming units (CFU)/mL achieved in under one hour and in complex samples [133]. In the case of sepsis in neonates, the limiting concentration of bacteria in the blood is 10 CFU/mL [138]. However, medical doctors are willing to wait only one hour before the administration of wide-spectrum antibiotics [139]. Another challenge is to lower the limit of detection to around 1 CFU/100 mL, which is needed for the analysis of drinking water [140].

The best balance between LOD and the time of analysis was reported in 2020 by Farooq and coworkers, who were able to detect 3 CFU/mL within 30 min. The authors used high-density layers of phages immobilized onto cellulose for the detection of *Staphylococcus aureus*, employing differential pulse voltammetry [141]. A similar solution was shown by Xu et al. [142], who achieved 14 ± 5 CFU/mL of *Escherichia coli* within 20 min, also using differential pulse voltammetry. Sedki et al. [143] achieved 14 CFU/mL of E. coli after 30 min of incubation using virions chemisorbed on a glassy carbon electrode decorated with gold nanoparticles and employing electrochemical impedance spectroscopy. Carboxyl graphene–PaP1 phages composite drop-casted onto the glassy carbon electrode allowed for the detection of *Pseudomonas aeruginosa* with an LOD of 56 CFU/mL and within 30 min. The read-out was achieved using electrochemiluminescence [144]. All of the aforementioned cases capturing a bacterial cell by surface-bound virions resulted in local changes of electric properties, which were later detected through electrochemical methods. Such an approach not only favors but requires fast detection as after a few to a few tens of minutes, the captured bacteria are usually destroyed, and progeny virions are released. This issue might be overcome by using only parts of virions, e.g., receptor binding proteins (RBPs) [145,146].

The utilization of the molecular machinery of the host allows for the detection of (a) progeny virions, (b) bacterial metabolites, and (c) reporter molecules, which were encoded in the modified genetic material of the phage probe. In all these cases, the bacterial cell acts as a signal amplifier, allowing for low LOD, but in exchange for a prolonged time of analysis. The first phage-based method reaching the limit of detection of 1 CFU per 100 mL utilized such an approach and was reported in 2003. Neufeld et al. [147] used virulent phage typing combined with the amperometric detection of cell marker enzyme activity β-D-galactosidase, release upon cell disruption, to achieve 1 CFU per 100 mL detection from 6 to 8 h. In 2018, the Nugen group reported a different phage-based approach, which also allowed for an LOD of 1 CFU per 100 mL achieved within around 10 h. The authors developed a filtration-based method utilizing genetically modified T7 phages. The genes for the reporter enzymes, i.e., a luciferase and an alkaline phosphatase, were fused to genes for carbohydrate-binding modules specific to cellulose. Reporter phages were used to infect E. coli trapped on a cellulose filter. The binding moieties facilitated the immobilization of the reporter probes released from the target cells upon their lysis [148]. The Nugen group also showed a syringe-based sensor utilizing the same T7 genetically modified phages with luciferase fused with carbohydrate-binding modules to achieve 20 CFU per 100 mL, but the time of analysis was reduced down to 5 h, which is two times shorter compared to the filtering-based method [140].

Progeny virions are usually detected by means of qPCR. Recently, Anany et al. [149] showed an interesting example, where phages were printed onto paper strips using modified inkjet. A phage dipstick was used to capture and infect E. coli O157:H7, E. coli O45:H2, and *Salmonella* Newport in spinach, ground beef, and chicken homogenates, respectively, and the reported LOD was in the range from 10 CFU/mL to 50 CFU/mL in 8 h.

Time and LOD might also be balanced by introducing the pre-enrichment step. For instance, in the case of work by Laube and coworkers [150], the phagomagnetic separation of bacteria labeled with antibodies conjugated with horseradish peroxide allowed for an LOD of 19 CFU/mL in 2.5 h. When samples were pre-enriched for 6 h, the LOD decreased to around 1.4 CFU per 25 mL.

Bacteriophages were also used for the detection of other analytes, e.g., ions [45] or organic compounds (an example of the device is shown in Figure 3B) [112]. Not only phage-displayed peptides [45] but also whole virions were used. For instance, Armon and Kott reviewed the use of bacteriophages as indicators of pollution [151]. Based on the bacteriophage persistence in water and their related survival time to human viruses, it was proposed to use bacteriophages as a useful index of viral pollution. It was shown that coliphages might be used as indicators of airborne viruses and enteric viruses. Some phages are also indicators of recreational water quality or even fecal indicators. Very recently, Kim et al. [152] showed the utilization of bacteriophage-based colorimetric sensors for the detection of medical chemicals. Three variants of the M13 phage were used: *wt*, and two others that displayed specific peptides which changed the hydrophobic/hydrophilic balance of virions, respectively. Red, green, and blue colored pixels of the array were achieved by varying pulling speeds upon the preparation of phage films. A color pattern was formed as a unique response value to a given analyte. A similar colorimetric method was used to detect the volatile organic chemicals and endocrine-disrupting chemicals [112].

### 3.3. Phage-Based Scaffolds

The structure of phages allows the creation of templates or scaffoldings, which can be further used in multiple applications. The most promising and thus exploited are filamentous phages, i.e., Ff, f1, Ike, fd, Pf1, and M13. Their length (up to 800 nm) and width (down to 8 nm) are perfect for the synthesis of nanowires and the fibrous components of composites [99,153,154,155,156,157].

Recently, more attention has been paid to the use of bacteriophages as components of materials designed for tissue regeneration. To restore the integrity of irreversible tissue damage, caused by diseases or accidents, a transplant from either the host itself or other donors (including humans or animals) is performed. In order to reduce the waiting time for transplants, artificial materials are being designed. Such materials mimic a natural supporting matrix—the extracellular matrix (ECM)—on which new cells can properly migrate, differentiate, and proliferate [143,148]. The strategies showed that the size and different design of scaffold could positively affect vascularization in newly formed tissue. The most promising experiments combined selected scaffold, the application of tissue-specific therapeutic cells (i.e., stem cells), and bioactive molecules (e.g., cytokines and growth factors). The chosen sequences of polypeptides are used as a growth stimulant [158]. However, the cost of such therapy may be a massive obstacle. The new strategies exploit genetically engineered phages as an inseparable component of an artificial ECM (see Figure 3C) to suppress the diffusion of polypeptides [113,159]. The genome of a phage is modified to expose multiple desired sequences of polypeptides at the surface of the capsid. Researchers show that the presence of phages in the human body is bioneutral, which is encouraging for their application. The application of phage display substrates in scaffolds was already shown in many materials designed for the regeneration of different tissues (i.e., cartilage, bone, or neural) [113,160,161,162,163,164,165,166].

Phages can be used for designing surface-enhanced Raman scattering (SERS) sensors by implementing phage display with the noble metal-binding motif. SERS sensors are usually based on nanostructured silver or gold surfaces [167,168,169]. The nanoarchitecture of those metals enhances the electromagnetic field of excitation light, which amplifies the signal from the analyte. The efficiency of enhancement of a given SERS substrate depends on the number of those nanostructures and their arrangement in the material. The M13 phage with a genetically modified protein coating was used to develop a scaffold for silver-based sensors [170]. The arrangement of a filamentous phage coated with metal particles enables the production of fiber-like material where hot spots are located either at the end or at contact spots of nanowires. The structure of the sensor showed an interesting pattern which could be useful in other applications. However, SERS substrates demand a denser hot spots layer in commercial materials (i.e., SERSitive; Warsaw, Poland).

### 3.4. Bacteriophages in Electrochemistry, Energy Storage and Generation

Bacteriophages have become useful in the production of materials used for electronics, such as small and flexible batteries or energy-harvesting devices [99,100,155,156]. Interest in phage-based batteries increased after 2006 when the Belcher group used M13 as a template for nanowires made of cobalt oxide (Co_3_O_4_) [171]. Such a design of electrodes became widely popular among scientists in the next years of research, where cobalt oxide was replaced by a variety of materials (i.e., single-wall carbon nanotubes [99,102], silver nanoparticles [172], nickel or even sulfur [99,173]).

The M13-based template was also used to synthesize a nanostructured FePO_4_ cathode for sodium-based batteries [174]. The cathode scaffold was obtained by modifying the phage capsid with a single-wall carbon nanotube (SWCNT) binding peptide motif. The cathode, resembling a corn cob and composed of M13, SWCNT, and Fe nanoparticles, was used to produce the Na/Bio-FePO_4_–CNT battery with a discharge capacity equal to 166 mAh/g at a C/10 rate (1 C = 150 mA/g).

A similar strategy to augment the contact area can be used to produce anodes using a nanofoam M13 phage-based scaffold [101]. In the initial step, M13 phages with EEAE (glutamic acid, glutamic acid, alanine, glutamic acid) metal (i.e., Ni) binding motif were crosslinked using glutaraldehyde. The phage scaffold was then metalized in the deposition bath using hypophosphite as a reducing agent. The amount of NaH_2_PO_4_ used during this step allowed for control over different Ni–P alloy phase compositions (i.e., Ni_3_P, Ni_2_P, and Ni_5_P_4_). The final product was used in lithium battery, which showed that a virus-based template enables obtaining 677 mAh/g discharge capacity, which was reduced by 20% after 100 cycles.

The piezoelectric properties of phages (i.e., M13) results from uneven distribution of charges within the phage capsid structure. The uneven distribution of charges is even observed within single coat protein pVIII (positively charged amino acid residues at the end of C terminus and the negative ones near N terminus). Under mechanical stress, the phage capsid structure is deformed, which changes the distance between charges. The change in directional polarization generates an electric field. The properly prepared device can register and collect this energy in the form of current flow. This opens the possibility of assembling the layers of ordered bacteriophages or other nanostructures towards the production of flexible piezoelectric energy generators [100,175]. Currently, the electric outcome generated within such energy harvesters is still below commercial expectations. The highest efficiency was obtained by Lee’s group, which used devices based on the vertically aligned M13 phage with genetically engineered coat proteins (pVIII with YEEE (tyrosine, glutamic acid, glutamic acid, glutamic acid) and pIII with 6H (6 histidines) motif). The design was based on phages self-assembling on Ni–NTA (nitrilotriacetic acid)/Au electrode using a polydimethylsiloxane (PDMS) mold. After the phage deposition, the surface was exposed to UV-light to crosslink the peptide residues between the phages. This approach stabilized the geometry of phages and their electrical and mechanical properties, which enhanced the piezoelectric effect. Finally, the energy harvester was formed by covering the phage layer by the second gold electrode. A combination of five such devices was enough to turn on the backlight of the LCD panel just by force from the pressing of the finger [99].

Perovskites are still gaining interest in the material science community. An innovative solution for increasing the packing of perovskite grains was presented in a work published by Hao-Sheng et al. [176]. The aim of the study was to create a solar cell in which the perovskite layer used the connection between the building grains with the help of M13 phages. The obtained material allowed for the increase in the efficiency of the solar cell from 17.8% to 20.1%. It is noteworthy that the connection with the lead perovskite took place without the need for the genetic modification of the phage. This shows that the phages do not have to act as a scaffold but can act as an adhesive element, which is possible to be heated to 90 °C without breaking the material. Following a similar approach, optoelectronic organic solar cells and the organic light-emitting diode was proposed by Lee et al. [177]. The authors exploited the ability of genetically modified bacteriophages to bind Ag/Au nanoparticles. The created scaffold consists of various noble metallic particles, which allowed for prominent localized surface plasmon resonance. The layer of the created biocomposite was directly applied as an element of solar cells and the diode. The material showed better photophysical properties in both devices; power conversion efficiency increased by 15.5% (for solar cell); threefold higher luminescence than the pristine devices (≈29,000 cd m^−2^ vs. 9000 cd m^−2^) was observed, and external quantum efficiency was increased by 22.6% (for the organic diode).

### 3.5. Bacteriophages in Catalysis

Irreversible transformation in the current century will change the standard methods of obtaining energy (i.e., burning fossil fuels), in favor of alternative energy sources. One of them is fuel cells—based on the electrochemical transformation of non-fossil fuels (i.e., ethanol, methanol, glucose, hydrogen) into electricity. The catalytic process often utilizes gold or platinum nanoparticles. To overcome power limitations (and increase efficiency), scientists increase the accessibility of active sites by remodeling the two-dimensional morphology of fuel cells into three dimensions. The metal-binding residues on the phage capsid were recently used to develop high-surface-area catalysts for fuel cells. Belcher exploited filamentous M13 scaffolds to prepare gold nanowires for carbon oxide electro-oxidation [178]. The obtained stability and high yield of the created cell allowed further improvement by covering the gold nanowire with platinum atoms. Such developed Au–Pt core-shell nanowires enabled carrying out the ethanol oxidation process, which significantly improved the perspectives of the application of such design. Authors designed cells with different atomic ratios of gold to platinum (2.6:1, 1.8:1, 1:1), which appeared to generate an up to 6.5 times higher steady-state current compared to a cell composed of only platinum (0.13 mA/cm^2^.) Four years later, Dunn’s group further developed the phage-based ethanol oxidation idea by binding gold nanoparticles covered with enzyme–glucose oxidase to phage-based scaffolds [179]. The combination of AuNP and enzyme molecules allowed the direct electron transfer in the designed biofuel cell. The achieved peak current of 1.2 mA/cm^2^ was higher compared to other works using similar effects.

Hernández-Gordillo et al. [98] presented the application of M13 phages to create porous TiO_2_ material. Titanium alkoxide and ethanol were mixed with phage suspensions. The mixture was calcinated at 500 °C. The final material had pores with an average size of 8.2 nm. Although the authors did not perform any photocatalytic studies, such templates based on either TiO_2_ or ZnO should be beneficial in terms of photocatalytic activity. The efficiency of photoelectrodes benefits from increasing the surface area by imposing porosity. M13 phage scaffolds have been introduced as an improvement of the technological process for the production of TiO_2_ photoelectrodes. The application of pure TiO_2_ is limited by the energy bandgap (3.2 eV and 3.02 eV for anatase and rutile structure, respectively). Efficient photoelectrodes are usually obtained by doping or combining TiO_2_ with other materials. Promising phage-templated photoelectrodes were prepared as hybrids with carbon nanotubes or noble metal (i.e., Au or Ag) nanostructures, showing the absorbance beyond 400 nm with a resonance band in visible spectra (ca. 550 nm) [156,180,181].

## 4. Bacteriophages in Soft Matter and Physical Chemistry

**Figure 4:**
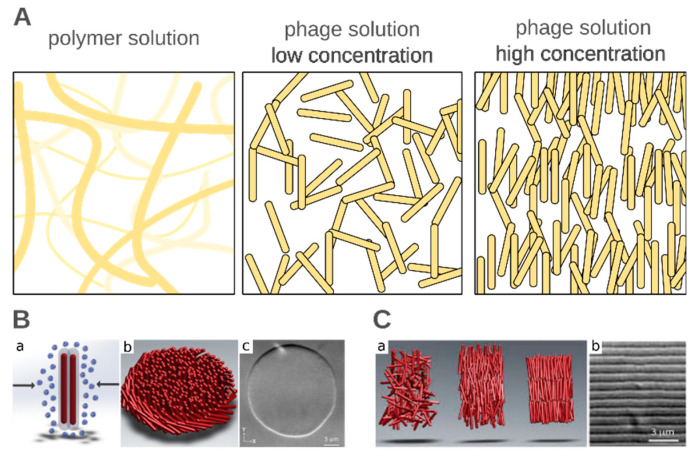
(**A**) Polymers usually form random coils at low concentrations, which interdigitate, creating meshes above the overlap concentration. However, phages, especially filamentous, are stiff colloidal rods. This property was used to study a number of physicochemical phenomena, e.g., transport and sedimentation, in an unusual crowded environment. In high concentrations, filamentous phage forms liquid crystalline phases. (**B**) (**a**) Non-adsorbing polymer causes attractive interactions between rod-like particles due to depletion. (**b**) The schematic representation of a 2D colloidal membrane composed of rod-like particles. (**c**) Differential interference contrast microscopy image showing a colloidal membrane formed by an fd phage. (**C**) (**a**) Cartoon showing the arrangement of rods in an isotropic, nematic, and smectic liquid phase. (**b**) Differential interference contrast microscopy image showing one-rod-length-long smectic layers. (**B**) and (**C**) are adapted from reference [157] based on the Creative Commons Attribution License (CC BY 4.0).

Bacteriophages play a valuable role in soft matter research. Phages are well defined soft materials of a variety of sizes and structures that form colloidal suspensions. The suspension of a small concentration of the selected phage (e.g., rod-like) dispersed in the solvent possesses isotropic properties, i.e., all virions are randomly oriented. However, upon an increase in concentration, phages can assemble into oriented anisotropic phases (i.e., liquid crystalline phases, see Figure 4). In the case of phage colloids, the system maximizes the entropy by lowering the number of states in which “phage-molecules” can subsist and increase the number of available states to the solvent molecules. Such behavior is observed and well defined as phase transitions in liquid crystals [157,182,183]. The possibility of the genetic modifications of the capsid proteins or changing the size of virions makes the phages an efficient toy model for analyzing or programming the self-assembly process. The highly concentrated colloids of filamentous or rod structure phages can produce not only one-dimensional orientation but also more complex phases as smectic, chiral, or even colloidal membranes [182]. Interesting patterns are observed when two types of different sized phages are mixed or when phages are mixed excluding the space polymers of high molecular weight. Understanding the interactions between virions and also between the main building components of phage—proteins, is necessary to produce even more complex and ordered structures. Sawada reviewed the virus-based soft materials obtained via controlled assembly through liquid crystalline formation [96]. Not only whole virions but artificial assemblies are also useful. Spakova et al. [184] extracted a phage tail tube building protein (gp39) from the phage NBD2 and recombined it in E. coli bacteria and yeast, *Saccharomyces cerevisiae*. After the incubation of bacteria, the protein was extracted and purified by the SDS-PAGE method. The phage protein tubes derived from both bacteria had a diameter of 12 nm and a length varying from 0.1 µm to more than 3.95 µm. The poly-protein tube was, on average, 23 times longer than the natural length of the phage (170 nm), which was caused by the absence of other capsid proteins that could regulate the building process.

The highly concentrated solutions of phages have also contributed to the research of the diffusion in complex liquids. The Brownian diffusion of molecules is a ubiquitous process in nature. The conventional diffusion equation defined by Einstein and Smoluchowski allows for the prediction of the mean distance and time of travel of a particle at given environmental conditions [185,186]. These models are the basis for the description of the reaction kinetics in diffusion-limited reactions. However, in the complex liquids, the diffusive motion of macromolecules fails to be described by standard models. The anomalous diffusion is attributed to macromolecular crowding along with the accompanying interactions (attraction and depletion) [187]. For example, the cell interior consists of a filamentous network that permeates the cell volume filled with differently sized proteins, lipids, and sugars. Multiple toy models of differently sized objects are used to resemble crowded environments at various configurations [188]. The application of phages as a crowding agent allowed for description of the diffusion in selected subsystems, where crowders possessed a monodispersed, rigid structure with chosen phase orientation (e.g., isotropic, nematic). To obtain new insights into the theory of diffusion, mostly filamentous phages are used in both experimental and computer simulation approach [189,190,191]. The experiments show that the isotropic suspensions of different concentrations of fd phage (7 nm × 890 nm) can highly affect the diffusion of proteins (apoferritin, hydrodynamic radius: 3.5 nm) [192,193]. The change of the diffusion coefficient is even more evident in the nematic phase, where it can be analyzed along or perpendicular to the orientation vector of virions.

When particle sizes become larger, diffusion under the influence of Brownian motion begins to be overpowered by the sedimentation process. The stability of monodisperse suspension is essential from the perspective of industrial applications, e.g., to extend the shelf life of food products. The influence of particle concentration, size, and external stress on the particle’s motion can provide valuable insights into the sedimentation process. This phenomenon was investigated by using non-interacting colloidal rods—fd and pf1 bacteriophages. In the work of Barabé et al. [194], the authors determined the correlation of reducing sedimentation as a function of the concentration of bacteriophages. By the application of two times longer rods, they observed the sedimentation speed decrease of two orders of magnitude compared to the same concentrations of shorter rods.

The highly concentrated suspension of fd phages (95 mg/mL) can organize into lamellar form. The formed stripwise pattern is repetitive, even at the macroscale. Alvarez et al. showed how the modified fd phage of different sizes (1.3 times bigger than used as crowder) diffuses in such a heavily complex system [154]. In this semi-crystalline system, diffusion is locally reduced from 3D to 1D, which is statistically the slowest type of diffusion (in terms of traveled distance). By the comparison of mean square displacement (MSD) determined from the trajectories of the labeled host and modified guest viruses, the authors showed that the guests’ diffusion was up to two times faster and much more prone to hopping-type diffusion (to the second column of phages/stripe).

Research on the formation of phage-based liquid crystal structures is not only a conceptual study, but examples of such constructs/formations are found directly in nature. P. aeruginosa is a bacterium that causes respiratory disease in humans—cystic fibrosis. The bacteria are infected by the Pf bacteriophage of rod-shaped structure [195]. Although bacteriophages are natural enemies of bacteria, the Pf phage contributes to a symbiotic-like relationship with bacteria [196,197]. During airway infections, bacteria tend to form a biofilm in which they multiply in the formed polymer matrix together with the phage. The rod-shape structured phage in high concentrations can order some of the space in the biofilm matrix into a crystalline structure. The research shows that bacteria that exhibit ordered biofilm possess higher resistance to antibiotics (i.e., tobramycin). This effect was explained by the formation of a denser polymer matrix capable of trapping antibiotic molecules. This not obvious symbiotic relation between phage and bacterium is responsible for morbidity increase and complicates the patient’s treatment.

Furthermore, the possibility of the genetic engineering or/and chemical modification of the capsid can significantly influence the physicochemical properties of the formed phases. Grafting polymers of different properties (e.g., thermoresponsive poly(N-isopropylacrylamide, PNIPAM) on a phage’s capsid affect the formation of standard liquid crystal phases and increase their stability [198,199]. The observations in the self-assembly process have already found beneficial applications in material sciences.

## 5. Concluding Remarks

This review shows that phages are gaining importance in various aspects of nanoscience. One could think that phages are useful only in applications when bacteria detection/identification is needed. In fact, phage therapies, biocontrol agents, sensors for bacteria detection, and antibacterial materials constitute important fields of study providing solutions for vital problems of the current world. However, phages proved to be useful and monodispersed building blocks and templates in nanotechnology, physical chemistry, and material science, due to their abundance corresponding to the variety of morphologies, robustness, ease of preparation, and ease of modification. The Nobel awarded phage-display technique caused an explosion of new and exciting examples of phage utilization.

The connection between nanotechnology and bacteriophages should be reciprocal: nanotechnology might and should allow for the modulation of the stability of bacteriophages. Even despite some phages are “tough”, some other species might lose activity under various physicochemical and physiological conditions. Phages are usually not selected based on their resistance to external factors, but rather due to their virulence, selectivity, ease of manipulation or modification. The recent review by Jończyk-Matysiak et al. [200] described factors determining phage stability, with emphasis on the effect of chemical substances, pH, freezing, heat, UV-light, and methods of preparation and formulation. This appears crucial, as for instance, in the case of the “*Phagoburn”* project, the patients received a very low dosage of phages (10^2^ pfu/mL daily). The titer of administered phages decreased by a thousand-fold within 15 days after production. A lack of stability was an essential factor causing the failure of the trial [83].

Different preservation methods, i.e., storage at 4 °C, freezing and storage at −80 °C or in liquid nitrogen, or the storage of dried or lyophilized phages, are used. Golec et al. proposed the method to store tailed phages [201] inside the infected cells at −80 °C without a major loss of phage and host viability. Immobilization [130] or encapsulation [202] was also showed to be efficient in increasing phage stability. Gonzalez-Menendez showed a comparative analysis of different preservation techniques for the storage of Staphylococcus phages [203].

Phage-based products might also take advantage of studies on the stabilization of eukaryotic viruses. The World Health Organization recognized the issue of protecting vaccines as one of the most critical challenges [204]. Nevertheless, only empirical solutions are available. For instance, the utilization of sucrose at molar concentrations for vaccine formulations has been in development [205,206,207]. Stewart et al. [208] demonstrated the preservation of adenovirus in the case of sucrose-stabilized liquid formulation for up to 6 weeks at 40 °C. Recently, a paper in Nature Communication coming from the Stellacci and Vitelli groups [209] described gold nanoparticles that improved the storage time of adenovirus type 5 at concentrations of several orders of magnitude lower compared to sucrose. Nano-based solutions for the stabilization of phages are yet to come.

On the other hand, phages are also enemies when they cause the contamination of bacteria-based biotechnological processes. Phage outbreaks may contribute to significant economic losses due to delays in processing and manufacturing, the poor quality of the product, material contamination, and eventually complete production loss [210]. Physical or chemical agents are used, but more versatile, safe, and user-friendly methods of the deactivation of bacteriophages are needed [200]. Nanotechnology gives a promise to provide a solution. Very recently, Richter et al. [211] found anti-phage nanoparticles that could be added directly into the bioreactors. The studied gold nanoparticles were effective against the T1, T4, and T7 phages. The reported decrease in titers was in the range from 2 logs within 5 h at 50 °C up to 5 logs after 24 h exposure. The combination of negatively charged and hydrophobic capping ligands was key for effective phage deactivation (inhibitory concentration of EC_50_ ≤ 1 µg mL^−1^).
